# Discordant lymphoma consisting of splenic mantle cell lymphoma and marginal zone lymphoma involving the bone marrow and peripheral blood: a case report

**DOI:** 10.1186/1752-1947-5-476

**Published:** 2011-09-23

**Authors:** Giovanni Carulli, Alessandra Marini, Eugenio M Ciancia, Joseph Bruno, Silvana Vignati, Paola Lambelet, Elisa Cannizzo, Virginia Ottaviano, Sara Galimberti, Francesco Caracciolo, Maria I Ferreri, Elena Ciabatti, Mario Petrini

**Affiliations:** 1 Department of Oncology, Transplants and New Technologies in Medicine, Division of Hematology and Section of Flow Cytometry, University of Pisa, Pisa, Italy; 2 Laboratory of Clinical Pathology, Versilia Hospital, Lido di Camaiore, Italy; 3 Division of Pathology 2, AOUP, Pisa, Italy; 4 Division of Pathology, Versilia Hospital, Lido di Camaiore, Italy; 5 Division of Medicine, Versilia Hospital, Lido di Camaiore, Italy; 6 Laboratory of Cytogenetics, AOUP, Santa Chiara Hospital, 56126 Pisa, Italy

## Abstract

**Introduction:**

Discordant lymphomas are rare entities characterized by the simultaneous presence of two distinct types of lymphomas in different anatomic sites. We describe a very rare case of simultaneous occurrence of splenic mantle cell lymphoma and marginal zone lymphoma involving the bone marrow and peripheral blood.

**Case presentation:**

We report the case of a 60-year-old asymptomatic Caucasian woman in whom discordant lymphomas were discovered when a slight lymphocytosis and a conspicuous splenomegaly were observed. The different morphological, immunophenotypical and immunohistochemical features found in the different pathologic samples obtained from peripheral blood, bone marrow and spleen sections made it possible to differentiate two types of non-Hodgkin B-cell lymphomas: a mantle cell lymphoma infiltrating the spleen and a marginal zone lymphoma involving both the bone marrow and peripheral blood. Since a similar IgH gene rearrangement was found both in the bone marrow and in the spleen, the hypothesis of a common origin, followed by a different clonal selection of the neoplastic lymphocytes may be taken into consideration.

**Conclusion:**

Our case emphasizes the usefulness of investigating simultaneous specimens from different anatomic sites from the same patient and the relevant diagnostic role of splenectomy.

## Introduction

The association of two distinct B-cell non-Hodgkin lymphomas in the same patient and involving different anatomical locations is a rare phenomenon. This peculiar presentation, called 'discordant lymphoma', has to be differentiated from the so-called 'composite lymphoma', which is the occurrence of two or more morphologically and immunophenotypically distinct lymphoma clones in a single anatomical site, that is within a single organ or tissue [1].

We describe a patient with mantle cell lymphoma (MCL) concomitant with a marginal zone lymphoma (MZL). The former was responsible for massive splenomegaly, while the latter was responsible for bone marrow infiltration and peripheral blood spread. A multidisciplinary approach was necessary for diagnosis, but splenectomy proved to be of particular relevance both for detecting the localization of MCL in our patient and for choosing the most appropriate therapy.

## Case presentation

A 60-year-old Caucasian woman who had a silent clinical history with the exception of obesity and mild diabetes had routine blood count and chemistry tests at the recommendation of her family doctor. A slight lymphocytosis (range: 4.0 to 4.5 × 10^9^/L) was observed. All the other blood parameters, including white blood cells, hemoglobin, hematocrit, platelet count, lactate dehydrogenase, plasma proteins, protein electrophoresis, and immunoglobulin levels, were within the normal range. Serum immunofixation was negative. Immunophenotyping of peripheral blood samples was carried out by flow cytometry using a FacsCanto II cytometer (Becton Dickinson) equipped with two lasers (488 and 633 nm). Samples (50 μL) were stained with fluorochrome-conjugated monoclonal antibodies (MoAbs) specific for the following antigens: CD3, CD4, CD8, CD5, CD16, CD56, CD19, CD20, CD22, CD23, FMC7, CD103, CD11c, CD25, CD10, CD38, CD45 (purchased from Becton Dickinson), and K and λ immunoglobulin light chains (purchased from Dako). A six-color panel was used for each tube, associating MoAbs conjugated with FITC, PE, PerCP-Cy5.5, PE-Cy.7, APC and APC-Cy.7. At least 200,000 events were acquired and data were processed using FacsDiva software (Becton Dickinson). Lymphocytes were gated using CD45 expression and right angle scatter. A second gate included CD19+ events and was used to analyze the expression of the other markers.

**Figure 1:**
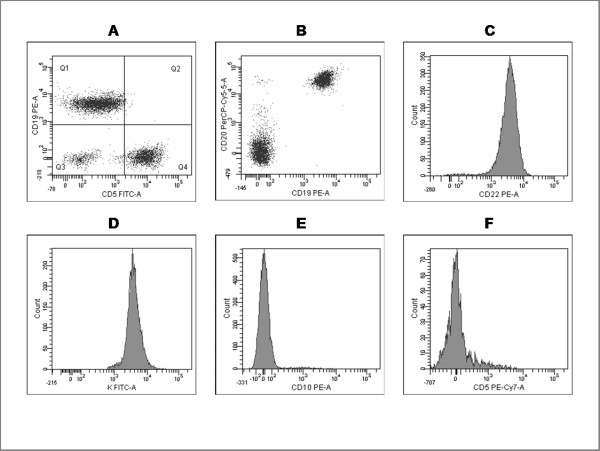
**Flow cytometry of peripheral blood lymphocytes**. CD19-positive lymphocytes are CD5- **(A)**, and CD20+ and CD22+ **(B, C)**, B-lymphocytes show restriction for surface K light chain **(D) **and are CD10-negative **(E)**. Negativity for CD5 is confirmed by the use of another anti-CD5 monoclonal antibody **(F)**.

Immunophenotyping showed an excess of B-lymphocytes (1.5 × 10^9^/L) with bright expression of CD20 and CD22, restriction for the K light chain of surface immunoglobulins, and absence of CD5 and CD10 (Figure 1). At light microscopy, lymphocytes were not villous (not shown).

A whole body computed tomography showed splenomegaly (20 cm longitudinal axis), but no lymphoadenomegaly or signs of other organ involvement were found.

The patient was referred to the Division of Hematology for further observation and underwent bone marrow evaluation (morphology by myeloaspirate specimens, trephine biopsy, flow cytometry, molecular biology assays and karyotype).

Bone marrow trephines were fixed in Myelodec^® ^reagent A (Bio-Optica) for two hours, decalcified in EDTA for two days, embedded in paraffin, and cut into 3 to 5 μm sections. Morphological evaluations were performed on hematoxylin-eosin, Giemsa and Gordon-Sweet for reticulin-stained sections. Immunohistochemical stainings were performed using a peroxidase-based system including antibodies specific for: CD20, CD3, CD5, CD23, DBA44, bcl2, bcl6, and cyclin-D1 (DSC-6). The spleen was sectioned and fixed in buffered formalin.

**Figure 2:**
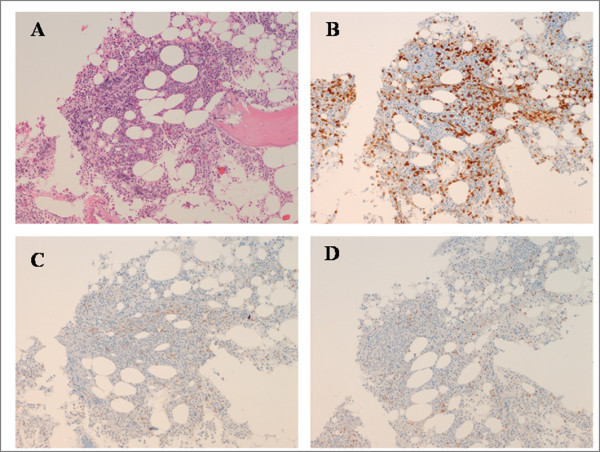
**Bone marrow histology**. A hematoxilin and eosin-stained section showing a discrete lymphocytic infiltrate, composed of small B cells as highlighted by anti-CD20 immunostaining **(B)**. Only a small percentage (fewer that 10%) of these cells are CD5-positive **(C) **or express cyclin D1 (D). The immunoprofile suggests a possible marginal origin for neoplastic lymphocytes. Magnification: 100×.

The bone marrow biopsy specimens showed a global cellularity of 50% with nodular-interstitial infiltration by CD20+ and bcl2+ lymphocytes, which accounted for 15% of cellularity and was negative for CD5, CD23, bcl-6, cyclin-D1 and DBA44 (Figure 2).

**Figure 3:**
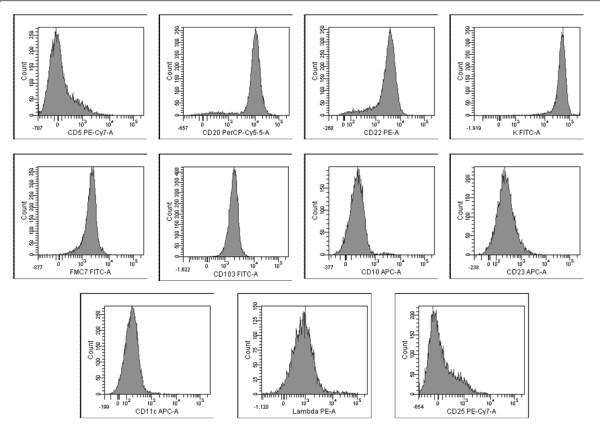
**Flow cytometric evaluation of bone marrow**. The neoplastic lymphocytes are positive for CD19, CD20, CD22, surface K light chain, FCM7, CD103, and negative for CD5, CD10, surface λ light chain, CD11c, CD25, CD23.

Flow cytometry of bone marrow blood showed 16% lymphocytes which were positive for CD19, CD20, CD22, CD103 and surface K light chain, and negative for CD5, CD23, CD10, CD11c, CD25, and surface λ light chain (Figure 3).

Mononuclear cells were separated by Ficoll/Hypaque gradient from bone marrow and peripheral blood samples, and suitable aliquots were utilized for polymerase chain reaction (PCR) tests after spectrophotometric quantitative evaluation. Fluorescent PCR reactions for IgH clonality evaluation were carried out with CDR3-specific VH consensus primer and analyzed by ABI PRISM 3100 (Applied Biosystems) [2]), whereas the results of FRI VH region rearrangement amplifications were run on a 3.5% agarose gel. Qualitative PCR detecting Bcl-1/JH rearrangements were performed according to the protocols established by the European network (BIOMED-2 Concerted Action) [3].

The molecular findings showed a single clonal rearrangement of the IgH gene and the absence of the bcl-1/JH translocation.

Karyotyping was carried out by conventional banding methods and no pathologic metaphases were detected by conventional karyotype.

Diagnosis of B-cell non-Hodgkin lymphoma, compatible with the MZL subtype, was therefore made.

The patient underwent splenectomy to remove a significant burden of disease and to confirm the initial diagnosis. Macroscopic examination showed that the organ was 23 × 15 × 8 cm, with a weight of 1350 grams. The spleen was sectioned and fixed in buffered formalin. Several samples were routinely processed to paraffin wax and sections of 3 to 5 μm were stained with hematoxylin-eosin for morphological evaluation. A panel of antibodies (CD20, CD3, CD5, CD10, CD23, bcl2, bcl6, cyclin-D1, CD10 and Ki-67) was applied to some samples using the ultraView Universal DAB Detection Kit and blue reagent with a BenchMark XT Automated Slide Stainer (Ventana).

**Figure 4:**
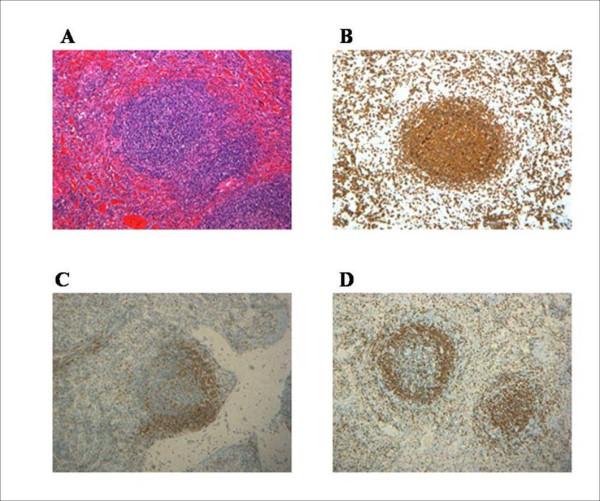
**Spleen sections**. **(A) **Expansion of the white pulp, which is composed of small or medium-sized elements showing variable morphology of the nucleus and pale cytoplasm. The red pulp is colonized and congested (H&E). **(B-D) **CD20, CD5 and Cyclin D1 expression. Magnification: 100×

**Figure 5:**
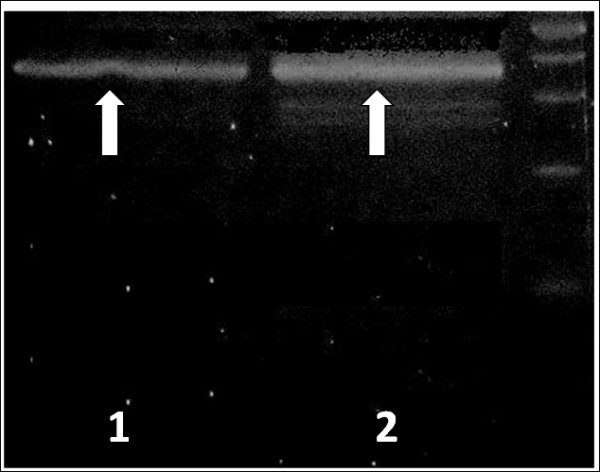
**Qualitative RT-PCR for IgH**. Rearrangement of IgH was assessed on the splenic sections (lane 1) and on the bone marrow (lane 2) with VH families-specific primers. Run on 3.5% agarose gel. The amplification pattern was the same (arrows).

**Figure 6:**
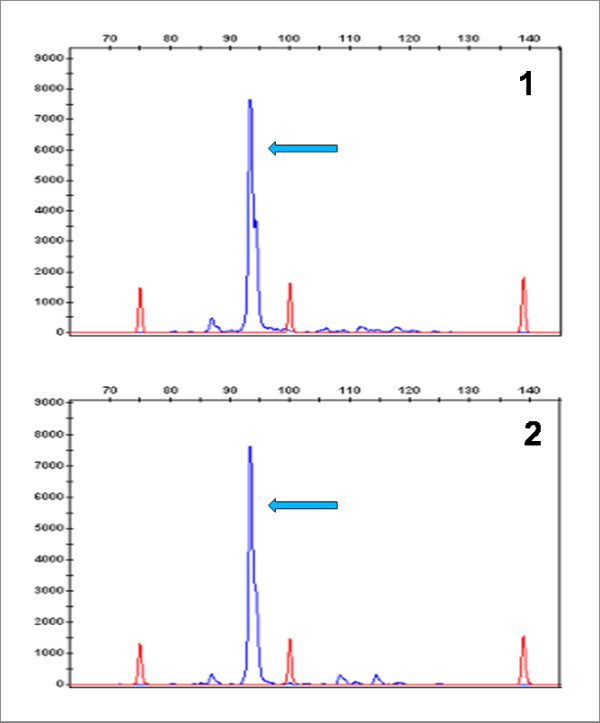
**Qualitative fluorescence PCR of IgH rearrangement**. Rearrangement of IgH was assessed on the splenic sections (lane #1) and on the bone marrow (lane 2). The length of the PCR product (93 bp) was the same in both organs (arrows).

The spleen was found to be infiltrated by a MCL; lymphocytes were positive for CD20, CD5, bcl2, negative for CD23 and CD10, had a low mitotic index (Ki-67: 5 to 10%), and showed a strong positivity for cyclin-D1 and a characteristic pattern of infiltration (Figure 4). The neoplastic lymphocytes were seen as small to medium-sized cells with irregular nuclear contours, giving rise to a mantle-fashion pattern of infiltration. DNA extracted from paraffin-embedded spleen specimens (EZ1 Advanced, Qiagen) was subjected to PCR assays, which showed a clonal IgH rearrangement, the same as found in the bone marrow (Figures 5 and 6).

At the end of our evaluation, the patient was diagnosed as suffering from the simultaneous presence of splenic MCL and bone marrow MZL with peripheral blood expression. Therapy with the cyclophosphamide, hydroxydaunorubicin (Adriamycin), Oncovin (vincristine) and prednisone - rituximab (CHOP-R) protocol was started and, after six courses and restaging by computed tomography and bone marrow and peripheral blood investigation, showed that complete remission was achieved.

## Discussion

Composite lymphomas involving a MCL and another type of B-cell non-Hodgkin lymphoma have sometimes been described [4], but the occurrence of discordant lymphomas seems to be rarer. In 2007 Goteri *et al. *[5] described the first case of a nodal MCL associated with a cutaneous follicular lymphoma.

We describe the case of a patient with discordant lymphoma characterized by the coexistence of splenic MCL and a CD5-negative non-Hodgkin lymphoma involving both the bone marrow and peripheral blood and compatible with a MZL.

The first pathologic finding in our patient was a very mild lymphocytosis with the presence of a clone of non-villous circulating B-cells, which were negative for CD5 and for bcl-1/JH translocation. A prominent splenomegaly was detected only after computed tomography, since the patient was asymptomatic and without any significant clinical history (with the exclusion of obesity). Tomography did not show nodal involvement or other sites suspected of disease localization.

The demonstration of a mild bone marrow involvement due to CD5-negative B-lymphocytes which were also CD103+, and negative for CD25, bcl-1/JH, and t(11;14), was consistent with a first hypothesis of splenic MZL. MZL are indolent B-cell diseases that putatively originate from the marginal zone of B-cell follicles, and can be found in the spleen, lymph node, and mucosal lymphoid tissues. Splenic MZL is a distinctive form of indolent lymphoma originating in the spleen, characterized by prominent splenomegaly and variable involvement of lymph nodes, bone marrow, peripheral blood, and other organs. Bone marrow infiltration is almost constant (83% to 100% of cases), while peripheral blood is involved in 29% to 75% in the various series [6]. MZL does not have a genetic signature. Unlike other types of non-Hodgkin lymphoma, such as follicular lymphoma and MCL, cytogenetic findings are not specific, so a diagnosis of MZL is made providing that other small B-cell lymphomas are excluded. The pattern of spleen infiltration provides the best diagnostic piece of information and splenectomy represents a very useful diagnostic tool. The immunophenotype of MZL lacks typical markers. CD5 might be positive in some cases, but the combination CD103+/CD25- has been suggested as a good diagnostic feature [7].

In our patient splenectomy was carried out both to confirm our provisional diagnosis and to remove a significant burden of disease. The spleen was infiltrated by MCL, with typical morphologic and phenotypic features (CD5+, cyclin D1+) [8].

Splenomegaly is a frequent finding in MCL (about 50% of cases) [9] and is usually associated with other disease localizations and variable outcome. Angelopoulou *et al. *[10] described a splenomegalic form of MCL with bone marrow infiltration and peripheral blood spread in which the infiltrating lymphocytes showed similar phenotypic features both in the spleen and in the bone marrow, and peripheral blood lymphocytes showed atypical morphology and the phenotype of MCL. The clinical course of such patients is more indolent than the common type of MCL and splenectomy is able to induce a partial regression of the disease.

In MCL, bone marrow involvement is a common finding, while a leukemic presentation is more variable and often associated with poor prognosis [11].

In our patient, the splenic findings, including morphology, immunophenotype and immunohistochemistry, were strongly consistent with MCL [8]. The clinical presentation of our patient, with asymptomatic disease and splenic involvement discovered accidentally, was very similar to the cases described by Angeloupolou *et al. *[10]. On the other hand, the characteristics of the neoplastic lymphocytes found in the bone marrow and in peripheral blood did not satisfy the common criteria for MCL [8], since they were CD5-negative, CD103-positive, cyclin-D1-negative, bcl-1/JH-negative and t(11;14)-negative. The most probable diagnosis was that of a simultaneous occurrence of a splenic MCL and a MZL, with the latter involving the bone marrow and peripheral blood.

An alternative diagnosis might be an association of MCL involving the spleen and an atypical MCL involving both bone marrow and peripheral blood.

Indeed, MCL shows immunophenotypic variations. It is known that some cases (about 10%) of MCL can be CD5-negative, or bcl-1/JH-negative (ranging from < 10% to 40%), or t(11;14)-negative (up to 50%) [12,13]. Cyclin-D1 positivity in the absence of CD5 expression makes a diagnosis of CD5-negative MCL more likely [14], due to the high diagnostic specificity of the former marker.

Thus, the simultaneous negativity for all the diagnostic markers of MCL seemed to exclude a diagnosis of MCL in the bone marrow. In addition, the immunophenotype of circulating lymphocytes was similar to that of bone marrow neoplastic cells.

The presence of the same IgH rearrangement both in bone marrow and in the spleen could suggest the hypothesis of a common origin of neoplastic lymphocytes, which might have undergone a different clonal selection, followed by the acquisition of two different phenotypes.

Therefore, the final diagnosis of a discordant lymphoma consisting of splenic MCL and bone marrow MZL with leukemic spread was established.

## Conclusions

Our case emphasizes the role of both the simultaneous investigation of specimens from different sites from the same patient and splenectomy in the diagnosis of cases of B-cell lymphomas with marked splenomegaly. In our patient, a wrong diagnosis would have been made without splenic histologic examination, leading to an erroneous therapy. In fact, splenic MZL is often treated with non-aggressive regimens including [15,16], while therapy of MCL involves more aggressive regimens, such as hyper-cyclophosphamide, vincristine, Adriamycin (doxorubicin) and dexamethasone (CVAD) or FCM, possibly associated with anti-CD20 MoAb [17]. MCL cases with marked splenomegaly but without nodal involvement can be treated with less aggressive regimens because of their more indolent behavior. We decided to use the R-CHOP schedule and a very good result was obtained, since disease restaging showed complete remission.

## Abbreviations

CHOP: cyclophosphamide; hydroxydaunorubicin (Adriamycin); Oncovin (vincristine) and prednisone; MCL: mantle cell lymphoma; MZL: marginal zone lymphoma.

## Consent

Written informed consent was obtained from the patient for publication of this case report and accompanying images. A copy of the written consent is available for review by the Editor-in-Chief of this journal.

## Competing interests

The authors declare that they have no competing interests.

## Funding

This study was supported by departmental grants.

## Authors' contributions

GC evaluated the patient and wrote the manuscript. AM, EC and VO carried out flow cytometry. EMC, JB and SV were the pathologists who examined the histological specimens. PL and FC evaluated and treated the patient. SG and MIF carried out PCR assays and cytogenetics. MP reviewed the manuscript. All authors read and approved the final manuscript.
